# The Role of Epsilon Near Zero and Hot Electrons in Enhanced Dynamic THz Emission from Nonlinear Metasurfaces

**DOI:** 10.1021/acs.nanolett.2c01400

**Published:** 2022-07-28

**Authors:** Eviatar Minerbi, Symeon Sideris, Jacob B. Khurgin, Tal Ellenbogen

**Affiliations:** † Department of Physical Electronics, School of Electrical Engineering, Tel-Aviv University, Tel Aviv 6997801, Israel; ‡ Center for Light-Matter Interaction, Tel-Aviv University, Tel-Aviv 6779801, Israel; § Raymond and Beverly Sackler Faculty of Exact Sciences, School of Physics & Astronomy, Tel-Aviv University, Tel-Aviv 6779801, Israel; ∥ Department of Electrical and Computer Engineering, Johns Hopkins University, Baltimore, Maryland 21218, United States

**Keywords:** epsilon near zero (ENZ), terahertz (THz), ITO, hot electrons, dynamic metasurface

## Abstract

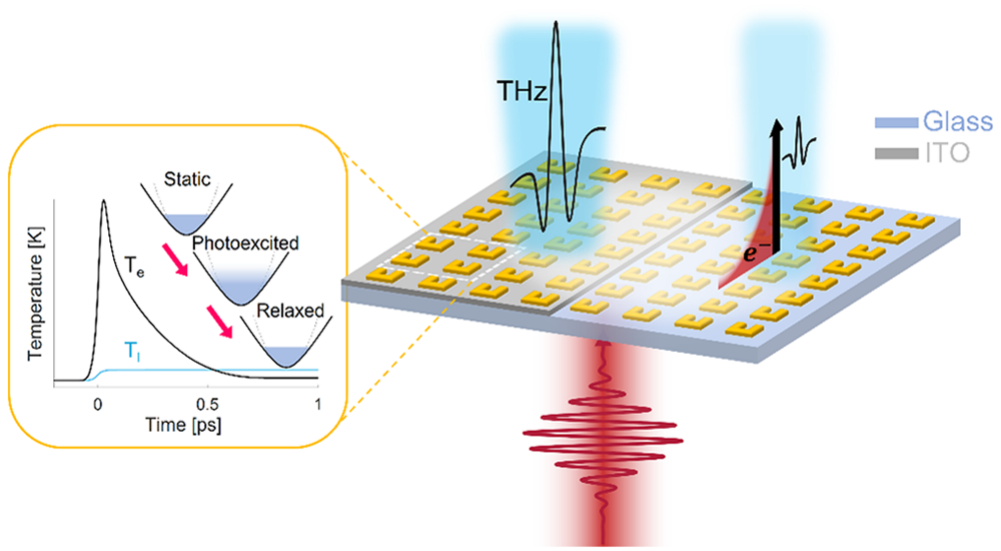

We study theoretically and experimentally the nonlinear THz emission from plasmonic metasurfaces and show that a thin indium-tin oxide (ITO) film significantly affects the nonlinear dynamics of the system. Specifically, the presence of the ITO film leads to 2 orders of magnitude stronger THz emission compared to a metasurface on glass. It also shows a different power law, signifying different dominant emission mechanisms. In addition, we find that the hot-electron dynamics in the system strongly modify the coupling between the plasmonic metasurface and the free electrons in the ITO at the picosecond time scale. This results in striking dynamic THz emission phenomena that were not observed to date. Specifically, we show that the generated THz pulse can be shortened in time and thus broadened in frequency with twice the bandwidth compared to previous studies and to an uncoupled system. Our findings open the door to design efficient and dynamic metasurface THz emitters.

Recently, surprisingly efficient THz emission following femtosecond laser excitation of nonlinear plasmonic metasurfaces has been reported.^1^ The magnitude of the field emitted from an ultrathin gold metasurface was shown to be comparable to that emitted from an orders of magnitude thicker zinc telluride (ZnTe) nonlinear crystal. Taking advantage of this effect, metasurfaces allowing phase control for generation of spatiotemporally tailored THz wavepackets have been demonstrated.^2−5^ However, the underlying physical mechanisms that enable such efficient THz emission are still not fully understood. Several processes, such as ponderomotive acceleration of photoejected electrons, either by multiphoton ionization or tunneling ionization, as well as optical rectification (OR) were proposed as the dominant mechanisms in the THz emission.^1,6−14^ Yet, a deeper understanding is still required to fully account for all the observations.

In many works that study THz emission from plasmonic metasurfaces, the metasurfaces are fabricated on thin ITO films, which are commonly used in the electron beam lithography process.^1−4^ Until recently, this layer was generally disregarded, and the nonlinear emission was considered to arise solely from the plasmonic nanostructures.^1,8,13,14^ However, the permittivity of ITO changes its sign from positive to negative in the near-infrared (NIR) region.^15−17^ It is also tunable and can be shifted up to the mid-infrared range by annealing in various atmospheric oxygen environments.^18,19^ This zero-crossing point coincides with the excitation wavelengths of some of the studied nonlinear metasurfaces and their resonant response. It was shown that at the epsilon near zero (ENZ) region, ITO as well as other materials possess strong optical nonlinearities and exhibit unusual properties, thus make promising candidates for new applications in both linear and nonlinear optics.^20−27^ A plethora of enhanced nonlinear effects were demonstrated in ITO films, such as second-harmonic generation (SHG),^28^ high harmonic generation,^29^ a nonlinear Kerr effect,^30^ and very recently also THz generation.^31^ However, since the amplification of the nonlinear effects is attributed to the enhancement of the normal component of the electric field at the ENZ region, they can only be observed when pumped at oblique incidence. To circumvent this constraint, hybrid metasurfaces constructed from plasmonic nanoantennas coupled to the ENZ material were designed and showed remarkably large second- and third-order nonlinearities.^5,32,33^

Here, we study the role of ITO and hot-electron dynamics in the THz emission from nonlinear plasmonic metasurfaces. To get better insight, we compare the emission from gold split ring resonator array (SRRs) metasurfaces, fabricated on a thin layer (∼20 nm) of ITO (referred to as SRR-ITO throughout this work) and on a bare SiO_2_ substrate (referred to as SRR-Glass). Figure 1a illustrates the unit cell structure. More details on the fabrication process along with SEM images of the fabricated samples are given in the Supplementary Notes 1 and 2.

**Figure 1:**
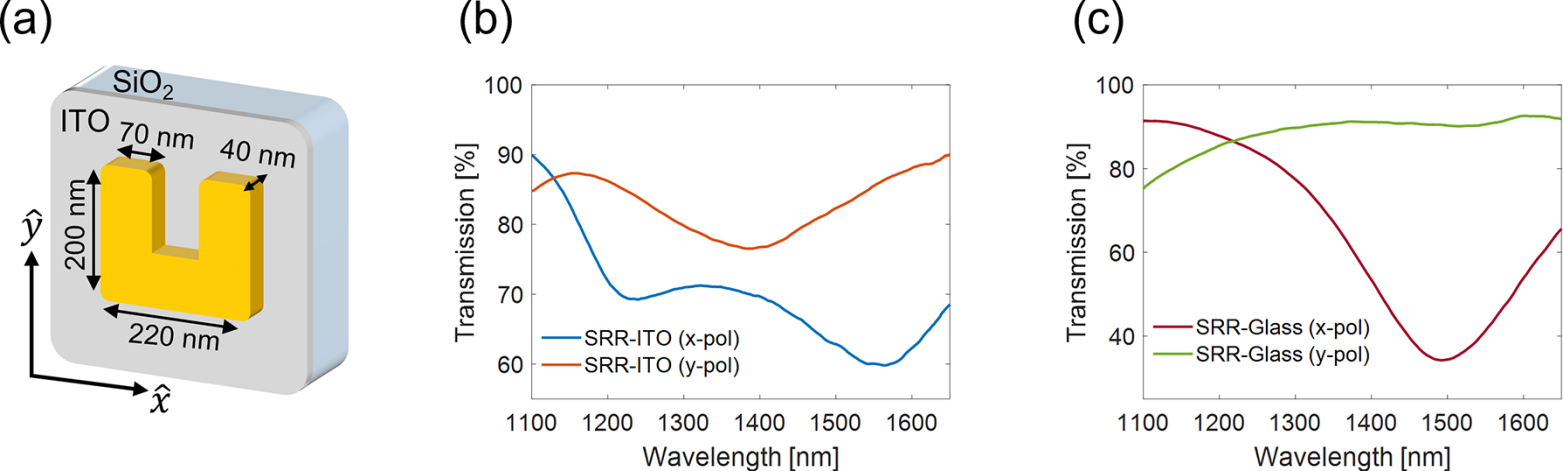
Metasurface characteristics. (a) Illustration and dimensions of a unit cell that composes the SRR-ITO metasurface. The dimension of the unit cell is 400 × 400 nm. The SRR-Glass metasurface is comprised of the same unit cell, except for the ITO layer. (b) Linear transmission of the SRR-ITO sample. Blue and orange lines represent *x̂*-polarized and *ŷ*-polarized illumination, respectively. (c) Linear transmission of the SRR-Glass sample. Red and green lines represent *x̂*-polarized and *ŷ*-polarized illumination, respectively.

We start by characterizing the linear response of the samples. Figure. 1(b,c) presents the polarized transmission spectra of the SRR-ITO and SRR-Glass samples, respectively. It can be seen that the SRR-Glass metasurface exhibits one resonance at λ_*res*_ ≈ 1500 nm when irradiated along the base of the SRRs (*E*_*in*_*x̂*) and no resonance when excited along the arms (*E*_*in*_*ŷ*). On the other hand, the SRR-ITO metasurface exhibits two resonances at 1240 and 1550 nm for *x̂*-polarized illumination and a single-resonance dip around 1400 nm for *ŷ*-polarized illumination. These results suggest that the nanoantenna modes of the SRRs are strongly coupled with the ITO mode. To further examine the purported coupling, we simulated the linear transmission as a function of the ITO thickness for different SRR dimensions (see Supplementary Note 2). These simulations indicate the strong coupling of the SRR mode and the ITO ENZ mode. The coupling is affected by both the widths of the arms and the ITO thickness. Therefore, by tuning the dimensions of the nanoparticles and the thickness of the ITO, it is possible to control the coupling of the system. For this reason, several previous works observed single-resonance transmission spectra, although the metasurfaces used were fabricated on ITO substrates^1,2^

Having described the linear properties of the metasurfaces, we turn to the nonlinear ones. We excite the metasurfaces with NIR femtosecond pulses (see Supplementary Note 3), to generate single-cycle THz signals. The emitted signal is characterized by a time domain spectroscopy system (TDS) based on electro-optic sampling (Figure 2a). THz emission is an even order nonlinear effect requiring breaking of inversion symmetry.^34^ It had been shown that due to the mirror symmetry along the base of the SRRs, the excitation configuration gives rise to nonlinear currents along the arms,^1,13,35^ and therefore, the emitted field is highly polarized along *ŷ*. Figure 2b,c shows the time- and frequency-resolved THz signal respectively, emitted from 1 × 1 mm^2^ uniform SRR-ITO and SRR-Glass metasurfaces, following pumping with 30 mW at a central wavelength of λ_*p*_ = 1500 nm. The emitted signal shows a single-cycle THz pulse with a pulse duration of about 1 ps. The signal peaks at ∼0.75 THz and extends to above 2.5 THz. It can be seen that the THz field generated from the SRR-Glass sample is significantly weaker than that of the SRR-ITO sample. This can be attributed to the strong nonlinearities of the ITO and field enhancement in the ENZ mode,^26,27^ as further discussed below.

**Figure 2:**
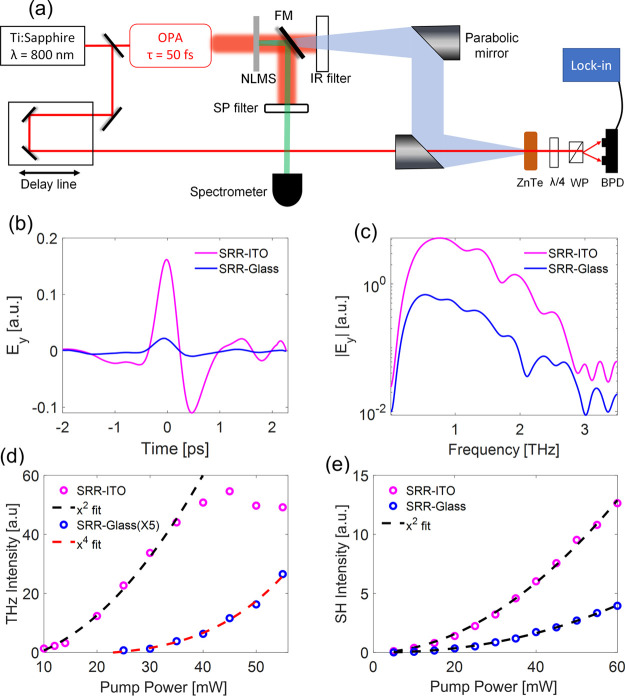
Nonlinear emission comparison between SRR-ITO and SRR-Glass metasurfaces. (a) Experimental setup. Ti:Sapp - Amplified titanium sapphire laser. OPA - Optical parametric amplifier. FM - Flip mirror. SP - Short pass. ZnTe - 0.5 mm (110-cut) crystal. WP - Wollaston prism. BPD - Balanced photodiode. (b) Time domain spectroscopy measurement of the THz signal emitted from SRR-ITO (magenta) and SRR-Glass (blue) under the same pumping conditions with an average power of 30 mW. (c) Emission spectrum from SRR-ITO and SRR-Glass metasurfaces. (d) Intensity of the THz signal generated from an SRR-ITO (magenta) and SRR-Glass metasurfaces (X5, blue) as a function of pump power. Dashed lines represent quadratic (black) and *x*^4^ (red) fits. (e) Intensity of SH signal generated from an SRR-ITO metasurface (magenta) and from an SRR-Glass metasurface (blue). Dashed black lines represent a quadratic fit.

The dependence of the generated THz intensity on the pumping power from the different samples reveals some unique properties, as shown in Figure 2d. First, it can be seen that the thin ITO film enhances the THz intensity up to 2 orders of magnitude (for a pumping power of 30 mW). In addition, the power law dependencies of the THz emission from metasurfaces on glass and ITO are different. This can be attributed to different dominant THz generation mechanisms in the two cases. The SRR-Glass sample shows a fourth power (*x*^4^) dependency, which can be explained by ponderomotive acceleration of photoejected electrons as was previously proposed.^8^ On the other hand, in the case of SRR-ITO, we observe a quadratic power dependence for up to ∼40 mW pump power. This suggests a second-order nonlinear process, optical rectification, as has been previously suggested,^1,12^ and settles the disagreement between the previous reports^1,8^ (see also supplementary of ref (1)).

At higher pumping power of the SRR-ITO sample, saturation is observed (Figure 2d). This saturation behavior is reversible and may point on dynamic effects involved in the generation process. To verify that these observations are unique to the THz emission, we also measured the second-harmonic generation from the samples. The results of the SHG measurements are shown in Figure 2e. In both samples, the intensity of the generated SH exhibits a quadratic dependence on the pump power, as expected. In addition, the SRR-ITO metasurface enhances the SH generation compared to SRR-Glass (up to ∼4 fold), which agrees with previous reports.^33^ This comparison shows that the ITO plays a more dominant role in the THz enhancement and may be explained by the large intrinsic nonlinearities arising from hot carriers in the THz regime that far exceed the fast nonlinearities in ITO.^26^

In order to explain the unique observations of the emission from the coupled SRR-ITO metasurface, we consider a nonlinear hydrodynamic model, which treats the electrons in the material as a fluid that obeys Euler’s equation.^14,35^ Using this model, we describe the second-order nonlinearity that arises in the metal nanoparticles as well as in the ITO layer (see Supplementary Note 4). The nonlinear currents generated by the OR act as the driving source of the THz emission.

Using this method, we are able to correctly predict the spectrum of the emitted field at low pumping powers, as presented in Figure 3a, as well as the quadratic power dependence (Figure 3b). We note that the spectrum predicted using this method does not depend on the pumping power and remains unchanged (thus referred to as “static model”). In addition, since this model only describes the OR process, it shows a quadratic power dependence and does not capture the saturation observed in Figure 2d.

**Figure 3:**
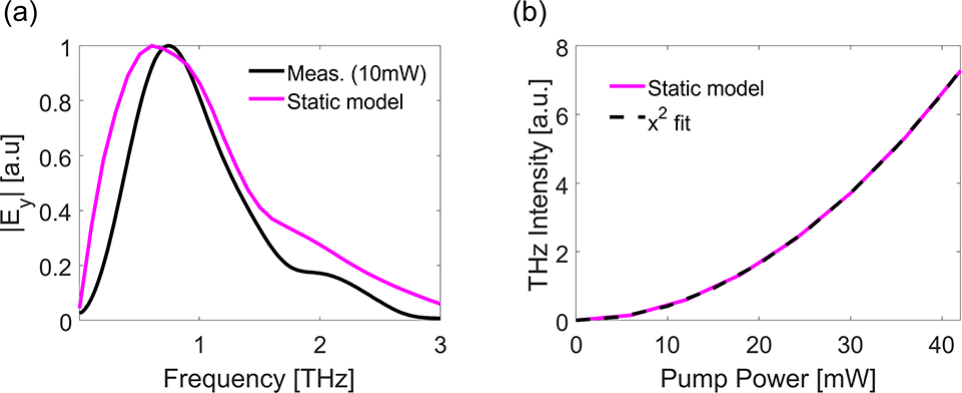
(a) THz emission spectrum measured for an *x̂* polarized pump at λ_*p*_ = 1500 nm with an average power of *P* = 10 mW (black), and the emission spectrum predicted by the static hydrodynamic model (magenta). (b) Intensity of THz signal as a function of pumping power simulated with the static model (magenta). The dashed black line represents a quadratic fit.

Moreover, the simulations of the system confirm that the strong THz emission recorded is due to the existence of the thin ITO layer rather than solely by the gold nanoparticles as was considered in previous studies.^13,14^ This large enhancement originates from the OR process in the ITO-SRR metasurface. The free carriers in the ITO are subject to strong asymmetric driving fields in the system, which are enhanced by field confinement, due to the SRRs and due to excitation wavelengths where the permittivity is near zero (see Supplementary Notes 5 and 6). Furthermore, the SRRs couple to the ITO to enable emission at normal incidence illumination.

Next, we examine more carefully the THz emission from the SRR-ITO sample. We see that pumping at either *x̂* or *ŷ* polarizations result in strong THz emission (see Supplementary Note 7). Figure 4 shows the generated signal when pumped with a fundamental wavelength of λ_*p*_ = 1300 nm (Figure 4a,c) and λ_*p*_ = 1500 nm (Figure 4b,d). The temporal and spectral shape of the pulse remains unchanged while pumping the weakly coupled resonance along *ŷ* (see Supplementary Notes 8 and 9). However, pumping the strongly coupled resonance along *x̂* results in shortening of the THz pulse and a broadening of the emitted THz bandwidth (Figure 4c,d). We measured a broadening of up to twice the bandwidth compared to previous studies and to the weakly coupled system (*E*_*in*_*ŷ*). In addition, pumping the strongly coupled system results not only in shorter pulses when increasing the pumping power, but the pulse shape changes as well. Also pumping at different wavelengths generates a different THz signal. This behavior may be explained by a phase difference between THz signals generated by short and long wavelengths (further explanation is given in Supplementary Note 8).

**Figure 4:**
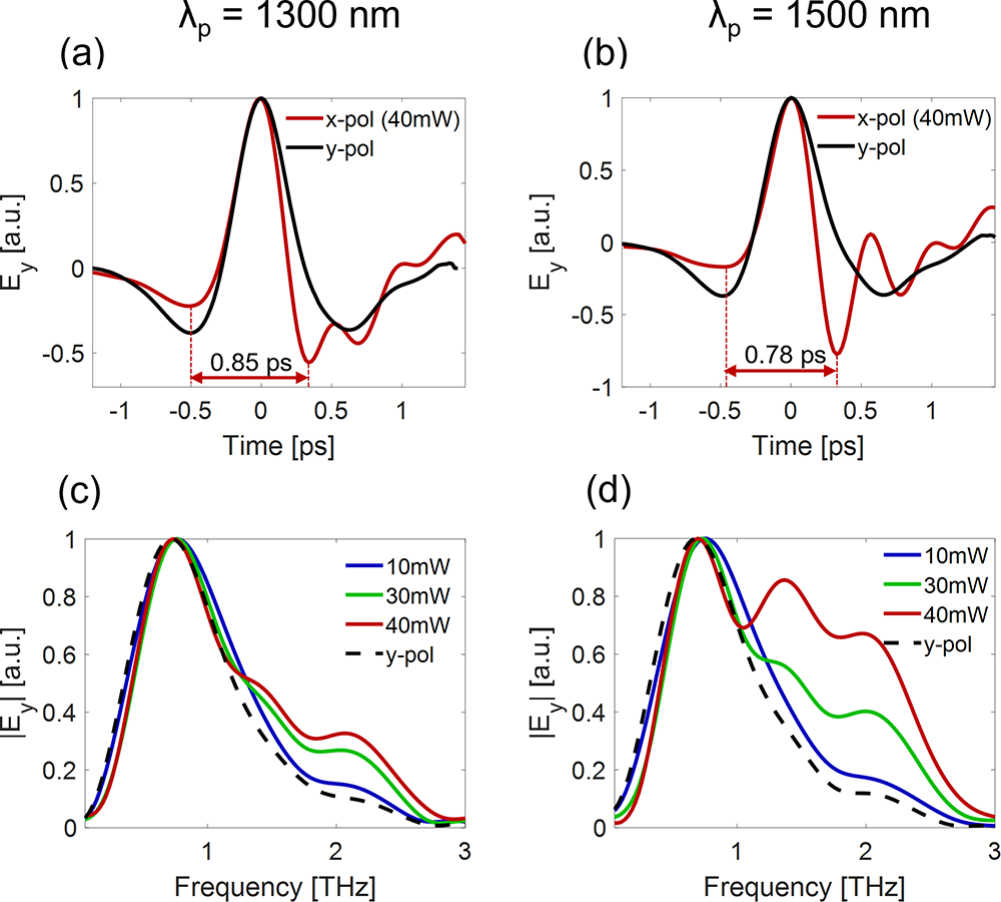
THz signal dynamics. (a) TDS measurements of the emitted THz signal when pumped at 40 mW with a wavelength of λ_*p*_ = 1300 nm and (b) of λ_*p*_ = 1500 nm. The red line represents pumping along the base of the SRRs (*x̂*-polarization), and the black line represents pumping along the arms (*ŷ*-polarization). The arrow marks the time of the THz cycle for *x̂*-polarized pumping. (c,d) Emission spectrum at different pumping powers for (c) λ_*p*_ = 1300 nm and (d) λ_*p*_ = 1500 nm for *P* = 10 (blue), *P* = 30 (green), *P* = 40 mW (red). The dashed black line represents the static THz spectrum generated by the weakly coupled system (*ŷ*-polarization).

To understand the saturation in the THz generated at high pumping powers and broadening of the spectrum, we take into account temporal dynamics that occur due to hot-electron generation in the ITO (see the schematic illustration in Figure 5a). The semiclassical two-temperature model (TTM) is used to calculate the spatiotemporal temperature distribution of the hot electrons in the gold nanoparticle and in the ITO layer together with their energy transfer to the lattice. In this model, the pumping NIR ultrashort laser excites the electrons, which then thermalize and also transfer the heat to the lattice. As a result of the fast electron and lattice heating, the effective mass changes due to the nonparabolicity of the conduction band. This leads to fast changes of the plasma frequency and the permittivity of the ITO. Therefore, the optical response is temporally changed at the sub-picosecond time scale. We account for this temporal thermo-optical change by altering the permittivity ϵ(*T*_*e*_, *T*_*l*_), which is dependent on the electrons (*T*_*e*_) and lattice (*T*_*l*_) temperatures (see Supplementary Notes 10–13 for more details on the theoretical model). These thermo-optical modifications dynamically change the coupling between the SRR resonance and the ITO ENZ mode, and therefore are evident for *x̂*-polarized excitation. Finally, the observed spectrum is highly dependent on the optical properties of the metasurface, which determine the frequencies that will radiate to the far field. Therefore, since the ultrafast heating process occurs at the subpicosecond time scale, the generated THz pulse is affected, thus resulting in the significant broadening of the emission spectrum. On the other hand, the generated SH is an almost instantaneous process and therefore remains unchanged by the delayed temporal dynamics. Using full wave simulations with a finite element method commercially available software^14^ accounting for these dynamics, we are able to reproduce the saturation behavior shown in Figure 2d. We show in Figure 5b that the quadratic power dependence of the THz emission observed at low pumping powers saturates at increasing powers when the heating effects become dominant. Saturation occurs due to a combined effect of a shift in the ENZ point^31^ and increase of the heated electron effective mass with pumping power, which reduces the strength of the ITO response due to a reduction in mobility. In addition, our framework also captures the broadening of the spectrum at increasing powers. Simulations of the generated THz spectrum when pumping the strongly coupled system are presented in Figure 5c,d and are in good agreement with the measurements (Figure 4c,d, respectively). Results for pumping the weakly coupled system are shown in Supplementary Note 9 and agree with the measured results as well.

**Figure 5:**
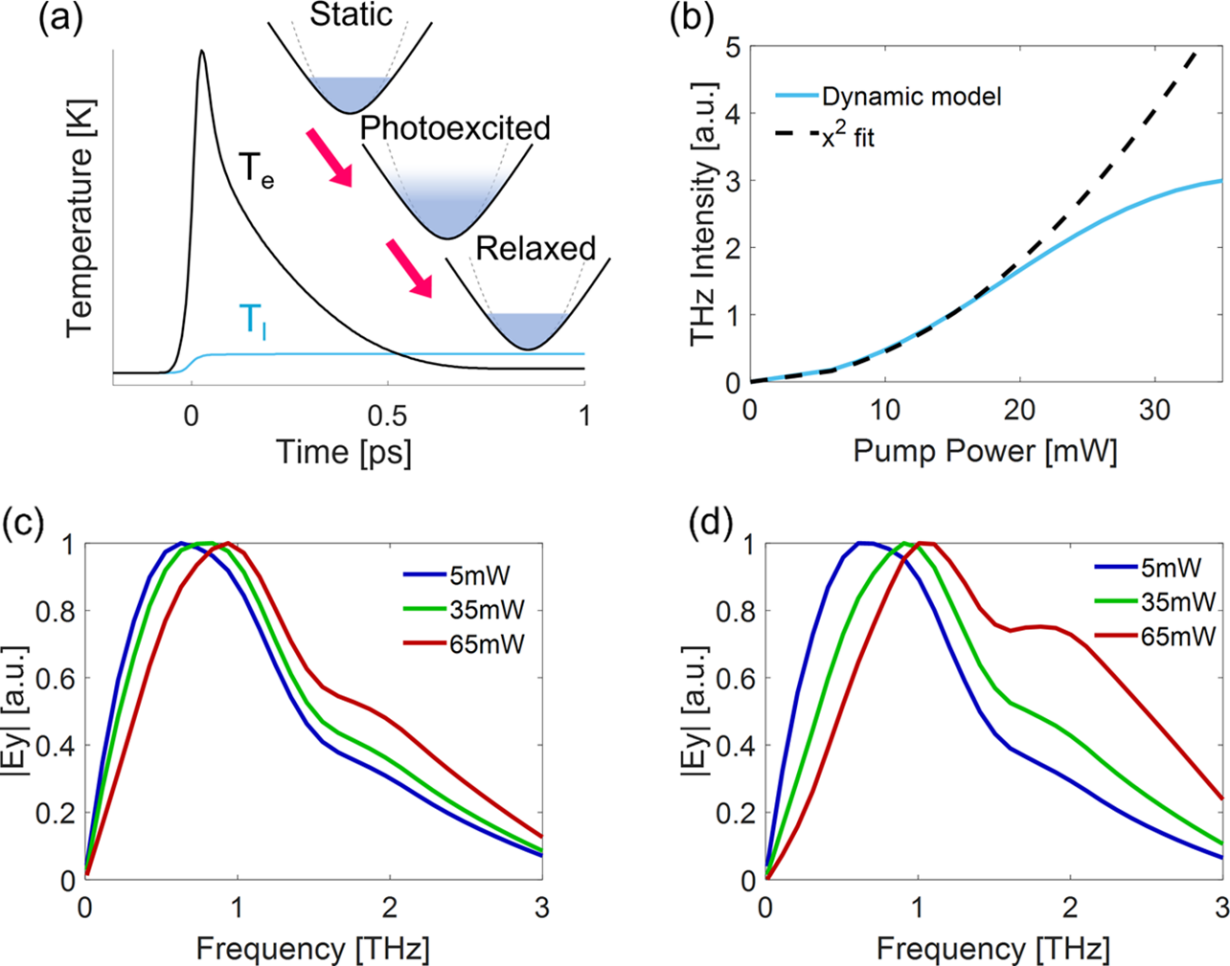
Theoretical model. (a) Illustration of the dynamical model. The ultrafast electron dynamics consist of photoexcitation by the IR pump pulse, hot-electron redistribution, thermalization by electron–electron scattering, and heat transfer to the lattice via electron–phonon scattering. The electron and lattice temperatures affect the optical properties of the system. The dynamic system then relaxes back to its static state in the picosecond time scale. The dashed line represents the parabolic conduction band. (b) Intensity of THz signal as a function of pumping power simulated with the dynamic model (light blue). The dashed black line represents a quadratic fit. (c) Simulated emission spectra for pumping wavelengths of λ_*p*_ = 1300 nm and (d) λ_*p*_ = 1500 nm at pumping powers of 5 mW (blue), 35 mW (green), and 65 mW (red).

In conclusion, we have shown that the strong THz emission from plasmonic metasurfaces is due to a thin film of ITO. This ∼20 nm thin layer enhances the THz emission by up to 2 orders of magnitude. In addition, we have shown that the strongly coupled SRR-ITO metasurface exhibits previously unreported dynamic phenomena. Specifically, broadening of the generated THz spectrum by a factor of 2 compared to previous reports and to an uncoupled system. To account for this behavior, we developed a dynamic theoretical framework which combines the hydrodynamic model as the source of the nonlinear THz emission with electron and lattice temperature-dependent permittivity. We see that our model agrees well the experimental results. These concepts unveil the fine fundamental physical dynamics of THz emission from nonlinear plasmonic metasurfaces. In addition, our work can advance the field toward efficient, active, integrated, and ultracompact optical elements for generating and controlling THz radiation.

## Supporting Information Available

(1) Sample fabrication method; (2) Linear response; (3) Measurement setup: (4) Hydrodynamic model; (5) Field enhancement in ITO layer; (6) SRRs and ITO contribution to THz generation; (7) THz dependence on pump polarization; (8) THz dynamics–time domain; (9) THz dynamics–frequency domain; (10) ITO nonparabolicity parameters; (11) Temperature-dependent effective mass; (12) Two-temperature model; (13) Thermo-optic response (PDF)

## References

[ref1] Luo L.; Chatzakis I.; Wang J.; Niesler F. B. P.; Wegener M.; Koschny T.; Soukoulis C. M. Broadband Terahertz Generation from Metamaterials. Nat. Commun. 2014, 5, 3055 10.1038/ncomms4055. 24402324

[ref2] Keren-Zur S.; Tal M.; Fleischer S.; Mittleman D. M.; Ellenbogen T. Generation of Spatiotemporally Tailored Terahertz Wavepackets by Nonlinear Metasurfaces. Nat. Commun. 2019, 10 (1), 1778 10.1038/s41467-019-09811-9. 30992447 PMC6467996

[ref3] Minerbi E.; Keren-Zur S.; Ellenbogen T. Nonlinear Metasurface Fresnel Zone Plates for Terahertz Generation and Manipulation. Nano Lett. 2019, 19 (9), 6072–6077. 10.1021/acs.nanolett.9b01970. 31356744 PMC6746046

[ref4] McDonnell C.; Deng J.; Sideris S.; Ellenbogen T.; Li G. Functional THz Emitters Based on Pancharatnam-Berry Phase Nonlinear Metasurfaces. Nat. Commun. 2021, 12 (1), 30 10.1038/s41467-020-20283-0. 33397951 PMC7782718

[ref5] Lu Y.; Feng X.; Wang Q.; Zhang X.; Fang M.; Sha W. E. I.; Huang Z.; Xu Q.; Niu L.; Chen X.; et al. Integrated Terahertz Generator-Manipulators Using Epsilon-near-Zero-Hybrid Nonlinear Metasurfaces. Nano Lett. 2021, 21 (18), 7699–7707. 10.1021/acs.nanolett.1c02372. 34498876

[ref6] Welsh G. H.; Hunt N. T.; Wynne K. Terahertz-Pulse Emission Through Laser Excitation of Surface Plasmons in a Metal Grating. Phys. Rev. Lett. 2007, 98 (2), 26803 10.1103/PhysRevLett.98.026803. 17358631

[ref7] Welsh G. H.; Wynne K. Generation of Ultrafast Terahertz Radiation Pulses on Metallic Nanostructured Surfaces. Opt. Express 2009, 17 (4), 2470–2480. 10.1364/OE.17.002470. 19219150

[ref8] Polyushkin D. K.; Hendry E.; Stone E. K.; Barnes W. L. THz Generation from Plasmonic Nanoparticle Arrays. Nano Lett. 2011, 11 (11), 4718–4724. 10.1021/nl202428g. 22007706

[ref9] Polyushkin D. K.; Márton I.; Rácz P.; Dombi P.; Hendry E.; Barnes W. L. Mechanisms of THz Generation from Silver Nanoparticle and Nanohole Arrays Illuminated by 100 Fs Pulses of Infrared Light. Phys. Rev. B 2014, 89 (12), 125426 10.1103/PhysRevB.89.125426.

[ref10] Takano K.; Asai M.; Kato K.; Komiyama H.; Yamaguchi A.; Iyoda T.; Tadokoro Y.; Nakajima M.; Bakunov M. I. Terahertz Emission from Gold Nanorods Irradiated by Ultrashort Laser Pulses of Different Wavelengths. Sci. Rep. 2019, 9 (1), 3280 10.1038/s41598-019-39604-5. 30824828 PMC6397179

[ref11] Kadlec F.; Kužel P.; Coutaz J.-L. Study of Terahertz Radiation Generated by Optical Rectification on Thin Gold Films. Opt. Lett. 2005, 30 (11), 1402–1404. 10.1364/OL.30.001402. 15981547

[ref12] Tal M.; Keren-Zur S.; Ellenbogen T. Nonlinear Plasmonic Metasurface Terahertz Emitters for Compact Terahertz Spectroscopy Systems. ACS Photonics 2020, 7 (12), 3286–3290. 10.1021/acsphotonics.0c01012. 33363248 PMC7754514

[ref13] Fang M.; Niu K.; Huang Z.; Sha W. E. I.; Wu X.; Koschny T.; Soukoulis C. M. Investigation of Broadband Terahertz Generation from Metasurface. Opt. Express 2018, 26 (11), 14241–14250. 10.1364/OE.26.014241. 29877464

[ref14] Sideris S.; Ellenbogen T. Terahertz Generation in Parallel Plate Waveguides Activated by Nonlinear Metasurfaces. Opt. Lett. 2019, 44 (14), 3590–3593. 10.1364/OL.44.003590. 31305579

[ref15] Rhodes C.; Cerruti M.; Efremenko A.; Losego M.; Aspnes D. E.; Maria J.-P.; Franzen S. Dependence of Plasmon Polaritons on the Thickness of Indium Tin Oxide Thin Films. J. Appl. Phys. 2008, 103 (9), 093108 10.1063/1.2908862.

[ref16] Kim J.; Shrestha S.; Souri M.; Connell J. G.; Park S.; Seo A. High-Temperature Optical Properties of Indium Tin Oxide Thin-Films. Sci. Rep. 2020, 10 (1), 12486 10.1038/s41598-020-69463-4. 32719380 PMC7385179

[ref17] Ali M.; Shehata A.; Ashour M.; Tawfik W. Z.; Schuch R.; Mohamed T. Measuring the Nonlinear Optical Properties of Indium Tin Oxide Thin Film Using Femtosecond Laser Pulses. J. Opt. Soc. Am. B 2020, 37 (11), A139–A146. 10.1364/JOSAB.396327.

[ref18] Wang Y.; Overvig A. C.; Shrestha S.; Zhang R.; Wang R.; Yu N.; Dal Negro L. Tunability of Indium Tin Oxide Materials for Mid-Infrared Plasmonics Applications. Opt. Mater. Express 2017, 7 (8), 2727 10.1364/OME.7.002727.

[ref19] Johns B.; Puthoor N. M.; Gopalakrishnan H.; Mishra A.; Pant R.; Mitra J. Epsilon-near-Zero Response in Indium Tin Oxide Thin Films: Octave Span Tuning and IR Plasmonics. J. Appl. Phys. 2020, 127 (4), 043102 10.1063/1.5128873.

[ref20] Bohn J.; Luk T. S.; Tollerton C.; Hutchings S. W.; Brener I.; Horsley S.; Barnes W. L.; Hendry E. All-Optical Switching of an Epsilon-near-Zero Plasmon Resonance in Indium Tin Oxide. Nat. Commun. 2021, 12 (1), 1017 10.1038/s41467-021-21332-y. 33589641 PMC7884677

[ref21] Reshef O.; De Leon I.; Alam M. Z.; Boyd R. W. Nonlinear Optical Effects in Epsilon-near-Zero Media. Nat. Rev. Mater. 2019, 4 (8), 535–551. 10.1038/s41578-019-0120-5.

[ref22] Minn K.; Anopchenko A.; Yang J.; Lee H. W. H. Excitation of Epsilon-near-Zero Resonance in Ultra-Thin Indium Tin Oxide Shell Embedded Nanostructured Optical Fiber. Sci. Rep. 2018, 8 (1), 2342 10.1038/s41598-018-19633-2. 29402902 PMC5799369

[ref23] Alù A.; Silveirinha M. G.; Salandrino A.; Engheta N. Epsilon-near-Zero Metamaterials and Electromagnetic Sources: Tailoring the Radiation Phase Pattern. Phys. Rev. B 2007, 75 (15), 155410 10.1103/PhysRevB.75.155410.

[ref24] Capretti A.; Wang Y.; Engheta N.; Dal Negro L. Enhanced Third-Harmonic Generation in Si-Compatible Epsilon-near-Zero Indium Tin Oxide Nanolayers. Opt. Lett. 2015, 40 (7), 1500 10.1364/OL.40.001500. 25831369

[ref25] Kinsey N.; Khurgin J. Nonlinear Epsilon-near-Zero Materials Explained: Opinion. Opt. Mater. Express 2019, 9 (7), 2793–2796. 10.1364/OME.9.002793.

[ref26] Khurgin J. B.; Clerici M.; Kinsey N. Fast and Slow Nonlinearities in Epsilon-Near-Zero Materials. Laser Photon. Rev. 2021, 15 (2), 2000291 10.1002/lpor.202000291.

[ref27] Secondo R.; Khurgin J.; Kinsey N. Absorptive Loss and Band Non-Parabolicity as a Physical Origin of Large Nonlinearity in Epsilon-near-Zero Materials. Opt. Mater. Express 2020, 10 (7), 1545–1560. 10.1364/OME.394111.

[ref28] Rodríguez-Suné L.; Scalora M.; Johnson A. S.; Cojocaru C.; Akozbek N.; Coppens Z. J.; Perez-Salinas D.; Wall S.; Trull J. Study of Second and Third Harmonic Generation from an Indium Tin Oxide Nanolayer: Influence of Nonlocal Effects and Hot Electrons. APL Photonics 2020, 5 (1), 010801 10.1063/1.5129627.

[ref29] Yang Y.; Lu J.; Manjavacas A.; Luk T. S.; Liu H.; Kelley K.; Maria J.-P.; Runnerstrom E. L.; Sinclair M. B.; Ghimire S.; et al. High-Harmonic Generation from an Epsilon-near-Zero Material. Nat. Phys. 2019, 15 (10), 1022–1026. 10.1038/s41567-019-0584-7.

[ref30] Alam M. Z.; De Leon I.; Boyd R. W. Large Optical Nonlinearity of Indium Tin Oxide in Its Epsilon-near-Zero Region. Science (80-.) 2016, 352 (6287), 795–797. 10.1126/science.aae0330. 27127238

[ref31] Jia W.; Liu M.; Lu Y.; Feng X.; Wang Q.; Zhang X.; Ni Y.; Hu F.; Gong M.; Xu X.; et al. Broadband Terahertz Wave Generation from an Epsilon-near-Zero Material. Light Sci. Appl. 2021, 10 (1), 11 10.1038/s41377-020-00452-y. 33414366 PMC7790823

[ref32] Alam M. Z.; Schulz S. A.; Upham J.; De Leon I.; Boyd R. W. Large Optical Nonlinearity of Nanoantennas Coupled to an Epsilon-near-Zero Material. Nat. Photonics 2018, 12 (2), 79–83. 10.1038/s41566-017-0089-9.

[ref33] Deng J.; Tang Y.; Chen S.; Li K.; Zayats A. V.; Li G. Giant Enhancement of Second-Order Nonlinearity of Epsilon-near- Zero Medium by a Plasmonic Metasurface. Nano Lett. 2020, 20 (7), 5421–5427. 10.1021/acs.nanolett.0c01810. 32496801

[ref34] Boyd R. W. Nonlinear Optics, 4th ed.; Academic Press, 2020.

[ref35] Ciracì C.; Poutrina E.; Scalora M.; Smith D. R. Origin of Second-Harmonic Generation Enhancement in Optical Split-Ring Resonators. Phys. Rev. B 2012, 85 (20), 201403 10.1103/PhysRevB.85.201403.

