# Supplementary material for: The Role of Epsilon Near Zero and Hot Electrons in Enhanced Dynamic THz Emission from Nonlinear Metasurfaces
Source: Nano Lett. 2022 Jul 28;22(15):6194–9. doi: 10.1021/acs.nanolett.2c01400 (PMC9373027; doi:10.1021/acs.nanolett.2c01400)
Supplement: Supplementary file 1 — nl2c01400_si_001.pdf [file nl2c01400_si_001.pdf]

Supplementary Information

The Role of Epsilon Near Zero and Hot Electrons in Enhanced Dynamic THz Emission from Nonlinear Metasurfaces

Eviatar Minerbi[1-3], Symeon Sideris[1,2], Jacob B. Khurgin[4], Tal Ellenbogen[1,2]

[1]*Department of Physical Electronics, School of Electrical Engineering, Tel-Aviv University, Tel Aviv, 6997801, Israel*

[2]*Center for Light-Matter Interaction, Tel-Aviv University, Tel-Aviv 6779801, Israel*

[3]*Raymond and Beverly Sackler Faculty of Exact Sciences, School of Physics & Astronomy, Tel-Aviv University, Tel-Aviv 6779801, Israel*

[4]*Department of Electrical and Computer Engineering, Johns Hopkins University, Baltimore, Maryland 21218, USA*

Author e-mail address: minerbi@mail.tau.ac.il

S1) Sample fabrication

Indium-Tin-Oxide (ITO) coated glass substrates were ordered from Sigma Aldrich. The ITO-coated glass and bare glass substrates were cleaned by sonication in acetone and isopropyl alcohol (IPA) and dried using a dry N2 stream. The clean substrates were spin-coated with polymethyl methacrylate (PMMA A4) and then baked at 180 °C on a hotplate for 1 min. The bare glass substrate was then spin-coated with electra-92 and then baked at 90 °C on a hotplate for 2 more min. The $1mm \times 1mm$ samples constructed from SRRs were written by an electron-beam lithography system (Raith 150-II) at 20 kV. The electra-92 was rinsed by immersing the bare glass substrate in purified water for 1 min. Both patterned substrates were then developed by immersing in cooled ($4°C$) MIBK/IPA 1:3 for 1 min, followed by drying under a dry N2 stream. A layer of 3 nm Ti was evaporated, followed by 37 nm Au. The remaining resist was lifted off in acetone and dried under a dry N2 stream. The SRRs had typically base lengths of ~ 220 nm, arm lengths of $\sim$200 nm, widths of ~75 nm, and a periodicity of 400 nm in a square lattice configuration (See Fig. S2 for SEM images).

S2) ITO linear response

Our sample was fabricated on a commercial ITO film with a sheet resistance of 70-100 Ω/sq deposited on a glass substrate. The thickness of the film is ~20nm and the thickness of the substrate is ~1.1mm. Since the film is highly doped, at near infrared wavelengths the optical properties of the ITO resemble a free electron gas. The near infrared permittivity of the ITO film was determined using ellipsometry measurements and fitting the data to the Drude model:

$$\epsilon_{ITO}(\omega) = \epsilon_\infty - \frac{\omega_p^2}{\omega^2 + i\omega\gamma}; \ \omega_p^2 = \frac{Nq^2}{\epsilon_0 m^*} \qquad (1.1)$$

where ω_p is the plasma frequency, γ is the scattering rate, and ϵ_∞ is the high frequency permittivity. m^* and q, are the electron effective mass and charge, respectively, N is the electron density, and ϵ_0 is the vacuum permittivity.

The values taken for the fit are $\omega_p = 3.07 * 10^{15} \left[\frac{rad}{s}\right]$, $\epsilon_\infty = 4.01$, $\gamma = 2.149 * 10^{14} \left[\frac{rad}{s}\right]$.

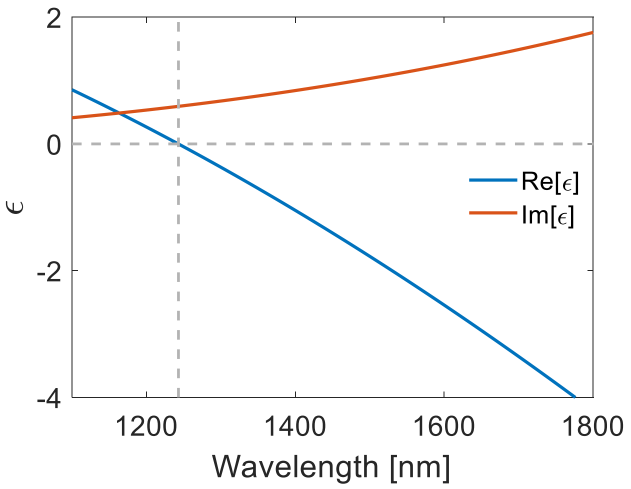

Figure S1: Real (blue) and imaginary (orange) parts of the permittivity of the ITO layer, retrieved from ellipsometry measurements. Dotted gray line marks the ENZ point at $\lambda_{ENZ} = 1245nm$

using these parameters, we simulate the transmission spectra for the SRR-ITO metasurface (MS), and for the SRR-Glass MS (Fig. S2).

The transmission spectra measurements were obtained using a supercontinuum white light laser source (SuperK COMPACT, NKT Photonics), and a polarizer. The transmitted light was then measured with a NIR spectrometer (NIRQuest, Ocean Optics)

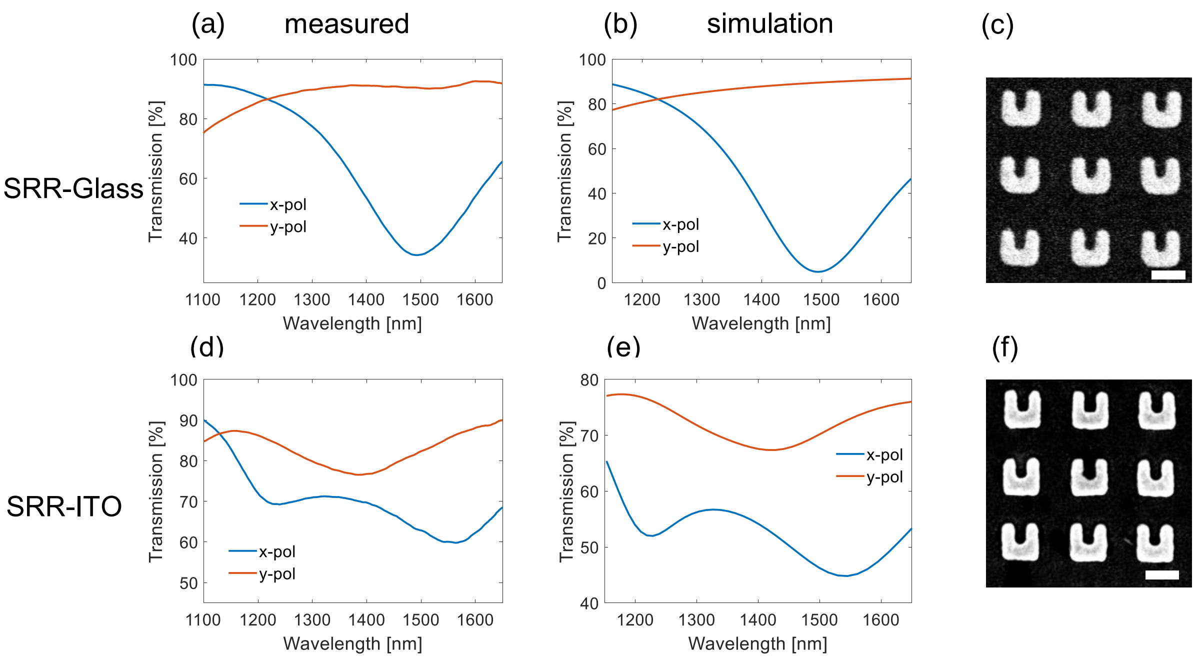

Figure S2: Measured (a,d) and simulated (b,e) linear transmission spectra of gold SRRs on glass (a,b) and on 20 nm of ITO (d,e). Orange line represents polarization along the arms of the SRRs (y polarization) and blue line represents polarization along the base (x polarization). c) SEM image of the SRR-Glass and f) SRR-ITO sample. White scale bar represents 200 nm.

To examine the purported coupling of the SRR mode and ITO confined bulk plasmon we simulated the linear transmission as a function of the ITO thickness for different SRR dimensions. The simulation results are shown in Fig. S3. It can be observed that the $\hat{y}$-polarized resonance redshifts when increasing the ITO thickness but is unaffected by the dimensions of the SRR (Fig. S3a-c). Therefore, this resonance can be attributed to the ITO ENZ mode. Even though this region is far from the $\hat{y}$-polarized resonance of the SRRs, the SRRs weakly couple the in-plane component of the incident field to the vertical field of the thickness-dependent ENZ mode. When exciting along the base of the SRRs ($E_{in}\hat{x}$), around the resonance of the SRRs, an avoided anti-crossing behavior is observed (Fig. S3d-f). This indicates the strong coupling of the SRR mode and the ITO ENZ mode.

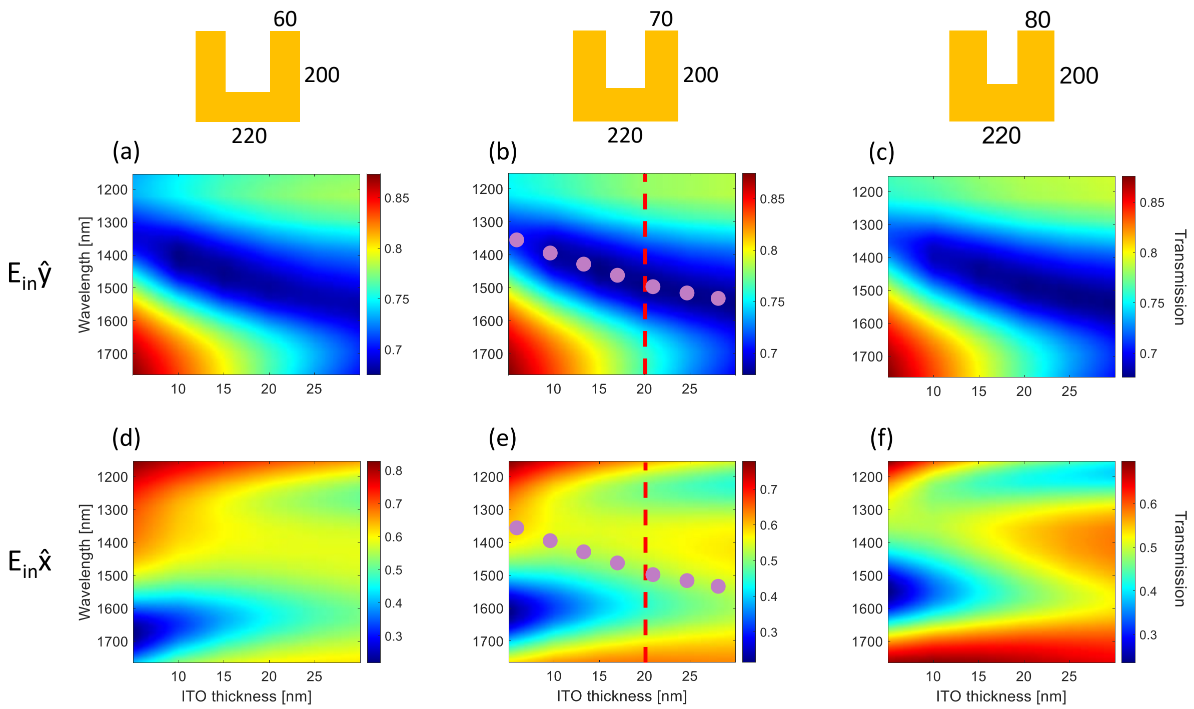

Figure S3: Linear transmission spectra of the SRR-ITO metasurface as a function of ITO thickness, for 3 arm widths: 60nm (a,d), 70nm (b,e) and 80nm (c,f). For $\hat{y}$-polarized (a-c) and for $\hat{x}$-polarized light (d-f). Purple circles represent the peak of the bulk plasmon resonance. Red line indicates the parameters used in this study

S3) Measurement setup

An ultrashort pulse (35 fs, 800nm central wavelength, 2kHz repetition rate, 3.5mJ pulse energy) emerging from a Ti:Sapphire amplifier (Spectra Physics Solstice ACE) passes through a beam splitter. The main portion of the beam is used to pump an optical parametric amplifier (OPA- Spectra Physics TOPAS) to generate the pump beam at varying NIR wavelengths with a pulse duration of about 50 fs. The power of the pump illuminating the NLMS is controlled using a half wave plate and a polarizer. A mechanical chopper chopped the pump beam at 1 kHz. The NIR beam illuminates the NLMS with average powers between 5-60mW (after chopper) and a beam diameter ~1.7 mm. The samples are placed such that the NLMS were facing the detector. For THz measurements, the residual transmitted NIR radiation is filtered out by a Teflon window. A small portion of the amplifier pulse is used as a probe for the THz signal by electro-optic sampling with a 0.5mm thick ZnTe crystal. The THz field induces birefringence in the crystal, which consequently rotates the probe polarization. A delay line is used on the optical axis of the probe, in order to temporally scan the THz pulse. The ZnTe crystal is followed by a quarter wave plate, a Wollaston prism and a balanced photodiode, in order to detect polarization modifications of the probe. The balanced photodiode signal due to the THz field is then detected by Lock-In amplifier (Stanford research systems SR830), coupled to a mechanical chopper located on the pump beam, which reduces the repetition rate on the NLMS to 1kHz. The delay line, scanning stages, chopper and lock-in amplifier were automatically controlled using LabVIEW.

Second harmonic (SH) measurements were taken by putting a flip mirror after the MS. The fundamental wavelength was filtered out by a short pass filter. The SH was measured with a spectrometer (Princeton Instruments FERGIE), and the intensity was calculated by integrating over the spectrum.

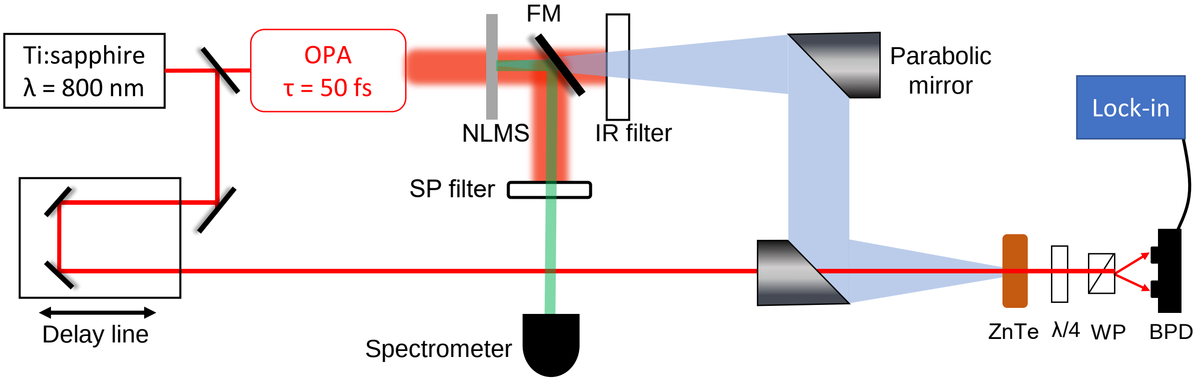

Figure S4: Experimental setup. Ti:Sapp - Amplified Titanium Sapphire laser. OPA - Optical parametric amplifier. FM -flip mirror. SP – short pass. ZnTe- 0.5mm (110-cut) crystal. WP- Wollaston prism. BPD – Balanced photodiode

S4) Hydrodynamic model

We follow previous works [1,2] and characterize the nonlinear optical response of the charge carriers in the gold and in the ITO using a hydrodynamic model of the electron gas. The hydrodynamic model is expanded perturbatively and we describe the bulk nonlinearities in terms of effective surface currents. Thus, expressing the second order nonlinearity in the MS by an equivalent surface current density.

In this model, the free electron motion induced by an electromagnetic field and the electron density, can be described by the Euler's equation:

$$\frac{\partial \boldsymbol{v}}{\partial t} + (\boldsymbol{v} \cdot \nabla)\boldsymbol{v} + \Gamma \boldsymbol{v} = \frac{e}{m^*}(\boldsymbol{E} + \boldsymbol{v} \times \boldsymbol{B}) - \frac{\beta^2}{N} \nabla N \qquad (4.1)$$

Together with the continuity equation:

$$\frac{\partial N}{\partial t} + \nabla \cdot (N\boldsymbol{v}) = 0 \qquad (4.2)$$

Where $\boldsymbol{E}$ and $\boldsymbol{B}$ are the electric and magnetic fields respectively, e and m^* are the electron charge and energy dependent effective mass respectively, $\boldsymbol{v}(\boldsymbol{r}, t)$ and $N(\boldsymbol{r}, t)$ are the electron velocity and density, respectively and β relates the electron pressure to the density within the Thomas-Fermi model and is proportional to the Fermi velocity[1]. The current is defined as $\boldsymbol{J} = eN\boldsymbol{v}$ and $\dot{\boldsymbol{P}} = \boldsymbol{J}$.

Neglecting the Lorentz force ($\boldsymbol{v} \times \boldsymbol{B}$) and nonlocal pressure effects in the free electron limit ($\beta = 0$) [1,2], we can expand the field perturbatively and get the expression for the nonlinear current density which act as a source for the THz field ($\omega_3 = \omega_2 - \omega_1$).

$$\boldsymbol{J_{nl}} = \frac{-i}{eN_0}\left[\omega_2(\nabla \cdot \mathbf{P_1})\mathbf{P_2^*} - \omega_1(\nabla \cdot \boldsymbol{P_2^*})\boldsymbol{P_1} + \frac{\omega_1\omega_2}{\omega_3 + i\Gamma}\left((\boldsymbol{P_1} \cdot \nabla)\mathbf{P_2^*} + (\mathbf{P_2^*} \cdot \nabla)\boldsymbol{P_1}\right)\right] \qquad (4.3)$$

Where, $\boldsymbol{P}_{1,2}$ is the polarization at frequency $\omega_{1,2}$. After simplification, the nonlinear surface currents can be expressed in terms of parallel and perpendicular components of the surface polarization.

$$\boldsymbol{K_{NL}} = -\frac{i}{eN_0}\left[\hat{\boldsymbol{t}}\left(\omega_1 P_2^{\perp *} P_1^{\parallel} - \omega_2 P_1^{\perp} P_2^{\parallel *}\right) + \widehat{\boldsymbol{n}}\left(\frac{1}{2}\omega_3 - \frac{\omega_1\omega_2}{\omega_3 + i\gamma}\right) P_1^{\perp} P_2^{\perp *}\right] \qquad (4.4)$$

This nonlinear current term can be applied to all geometries, however for symmetric shapes the overall current will cancel out. For geometries lacking inversion symmetry, such as SRRs (along the arms), the overall current is nonzero, leading to THz emission.

We note that the optical properties of the materials in the infrared as well as in the THz regime are important in order to correctly simulate the system.

S5) Field enhancement in ITO layer

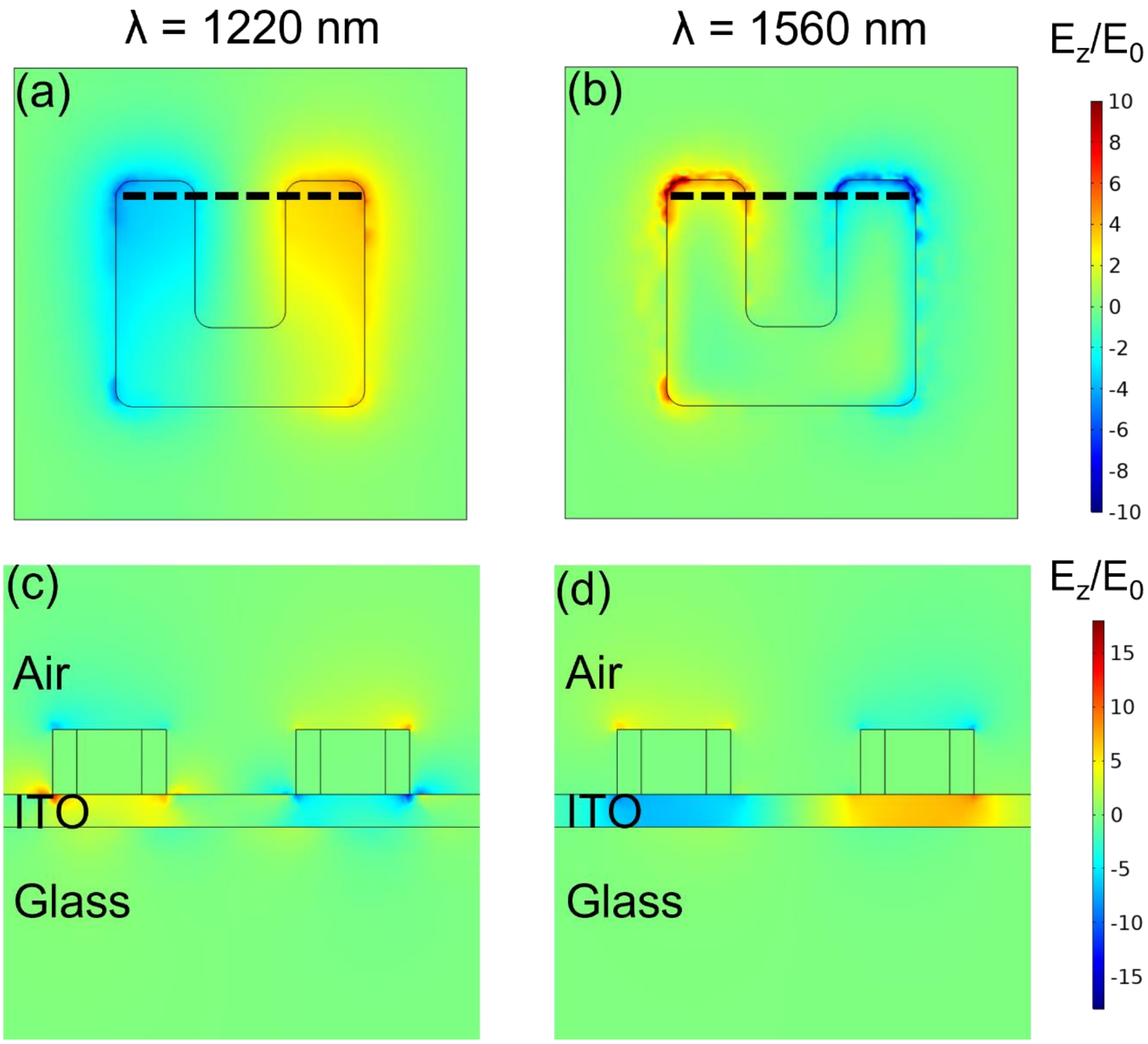

Figure S5: Field distribution: the plots show the field distribution (normal component) at the SRR-ITO interface (a,b), and inside the ITO along the horizontal black line (c,d). Left side (a,c) shows the field distribution of the resonance at the short wavelength ($\lambda \approx 1220$). Right side (b,d) shows the field distribution of the resonance at the long wavelength ($\lambda \approx 1560$).

S6) Contribution to THz generation

As shown in the main text, the THz generation mechanism for SRR on glass is different than SRR on ITO, which manifests in different power law dependencies (Fig. 2). In addition, the generated THz field from a SRR-ITO MS is an order of magnitude stronger than from SRR-Glass. Therefore, the THz is generated by OR in the SRR-Glass MS needs to be over an order of magnitude weaker than that of SRR-ITO. We investigate this by comparing three cases using the nonlinearity from the hydrodynamic model (OR).

First case, SRRs on a glass substrate, where the SRRs are the sole contributors to the nonlinear currents (presented as SRR-Glass in Fig. S6). Second case, SRRs on ITO, but the SRRs are still the only contributors to the nonlinear current and the ITO just enhances the field on the boundary between the ITO and the SRRs (presented as SRR-ITO FE in Fig. S6). Third case, SRRs on ITO and both the SRRs and the ITO contribute to the nonlinear currents (presented as SRR-ITO in Fig. S6). It can be seen that the THz generation by OR from the SRR-ITO MS is 4 orders of magnitude stronger than that generated from the SRR-Glass MS. The measurements shown in Fig.2 in the main text show only an order of magnitude difference in addition to fourth power dependency (OR has a quadratic power dependency). This fact further indicates that the leading contribution to the THz generation from plasmonic nanoparticles on a dielectric substrate can be attributed to ponderomotive acceleration rather than OR.

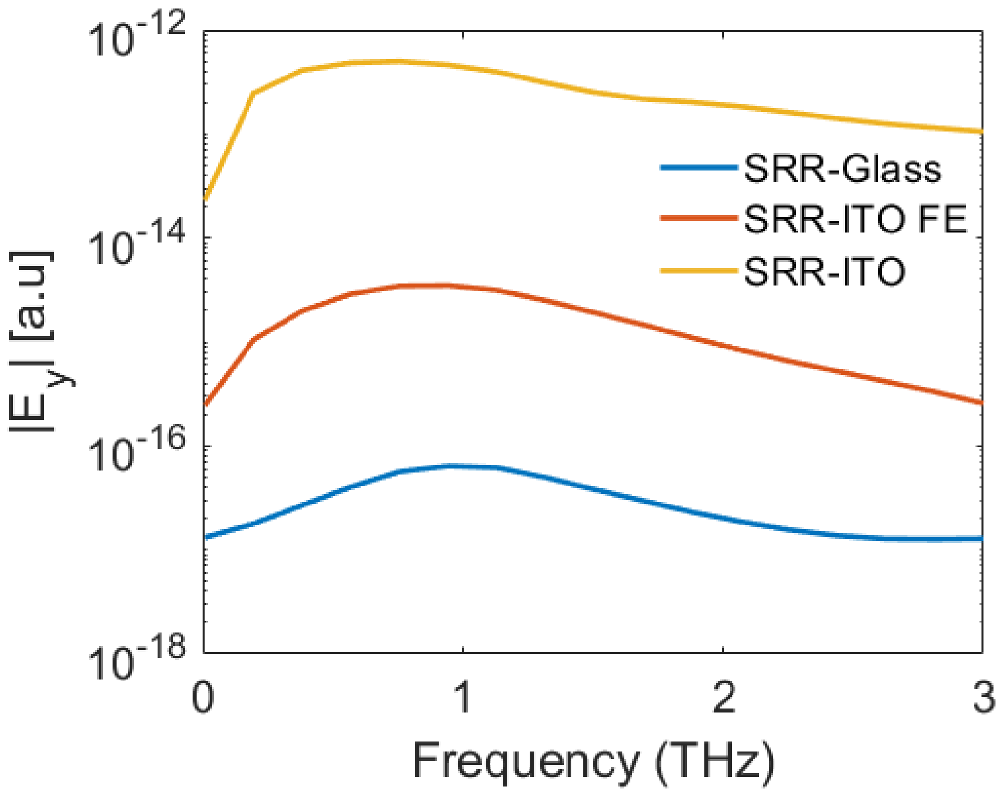

Figure S6: Emission spectrum from SRR-Glass (blue), SRR-ITO, field enhancement (FH) only (orange), SRR-ITO (yellow)

S7) THz dependence on pump polarization

Pumping at either $\hat{x}$ or $\hat{y}$ polarizations result in strong THz emission. When pumping the weakly coupled system (y-pol), slightly stronger THz emission is recorded compared to pumping the strongly coupled system (x-pol).

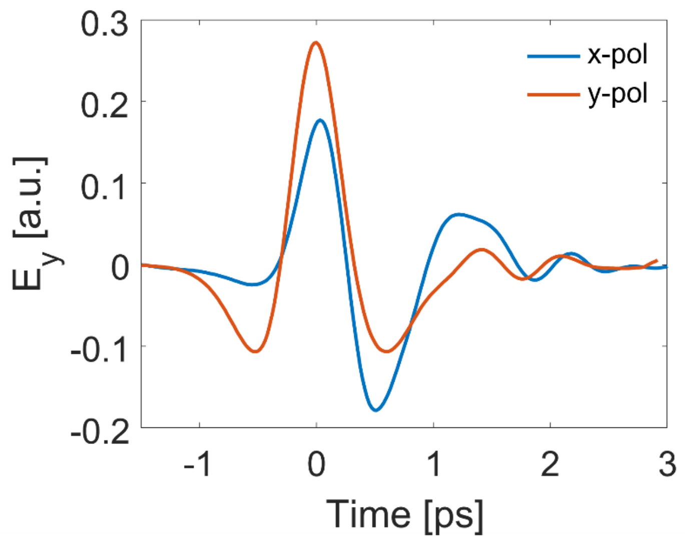

Figure S7: Time domain spectroscopy measurement of the THz signal emitted when pumping with 20mW at a central wavelength of $\lambda = 1500\ nm$, along $\hat{x}$ (blue) and $\hat{y}$ (orange).

S8) THz dynamics – time domain

Pumping the weakly coupled system, i.e exciting only the ITO plasmon mode generates a THz signal with a pulse shape that does not depend on the pumping power nor wavelength (Fig. S8).

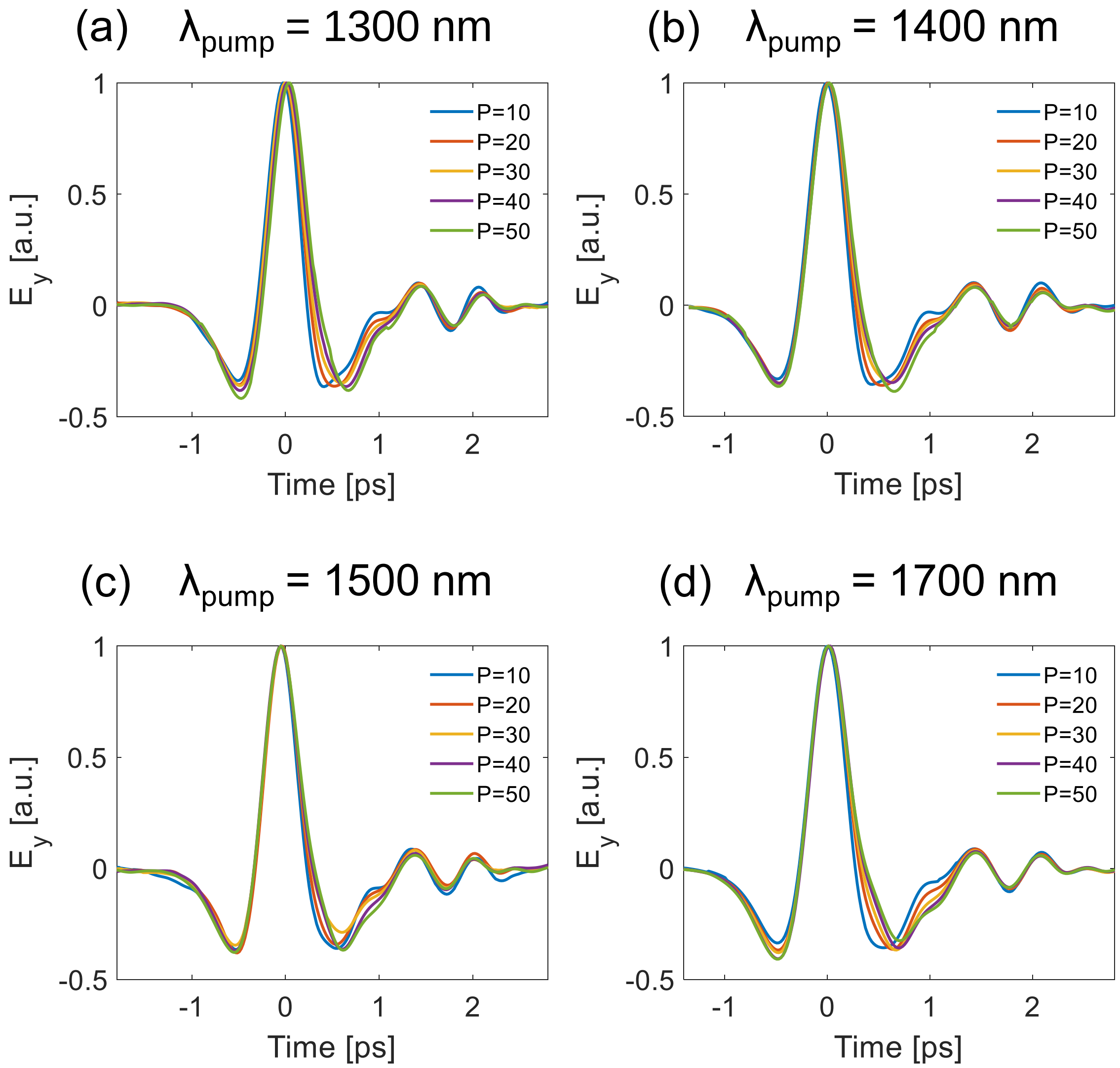

Figure S8: TDS measurements of the emitted THz signal at various powers when pumping the weakly coupled system ($E_{in}\hat{y}$). For pumping wavelengths of a) $\lambda_{pump} = 1300nm$, b) $\lambda_{pump} = 1400nm$, c) $\lambda_{pump} = 1500nm$, and d) $\lambda_{pump} = 1700nm$.

However, pumping along $\hat{x}$ results in a dynamic behavior that depends on the fundamental wavelength and on the pumping power. When pumping at the short wavelength resonance, the backside of the waveform gradually diminishes with increasing pumping powers (fig. S9a). On the other hand, pumping the long wavelength resonance results with an opposite behavior. In this case, the frontside of the initially symmetric pulse increases with increasing pumping powers (fig. S9b). To get further insight on this behavior we plot a signal and its

envelope for $\lambda_p = 1250$ and $\lambda_p = 1600$ in Fig. S9c,d respectively. We see that the change in the waveform is because of a group delay. The higher the pumping power, the faster the signal arrives (with larger group delay for low powers).

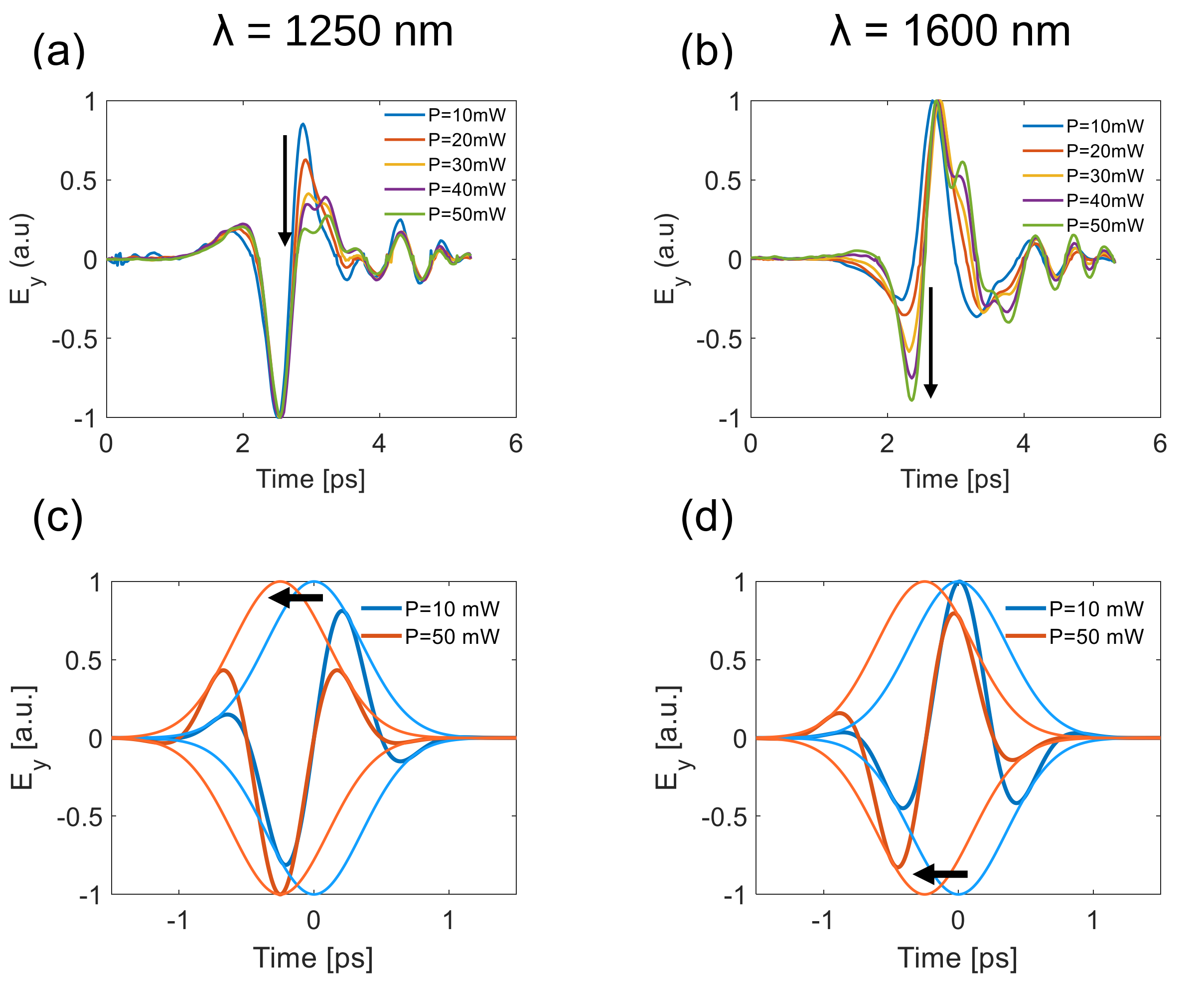

Figure S9: Time domain dynamics when pumping the strongly coupled system ($E_{in}\hat{x}$). a) TDS measurements of the emitted THz signal at various powers when pumped with a fundamental wavelength of $\lambda_{pump} = 1250nm$ and b) $\lambda_{pump} = 1600nm$. c) signal and envelope at pumping powers of P=10mW (blue) and P=50mW (orange) for $\lambda_{pump} = 1250nm$ and d) $\lambda_{pump} = 1600nm$. In all figures, the arrow shows the trend when increasing the pumping power.

S9) THz dynamics – frequency domain

When the pumping NIR pulse excites the weakly coupled ITO mode (y-polarization) the generated THz spectra is not affected, however exciting the strongly coupled SRR-ITO system (x- polarization) results in a broadening of the generated pulse. Longer pumping wavelengths exhibit increased broadening.

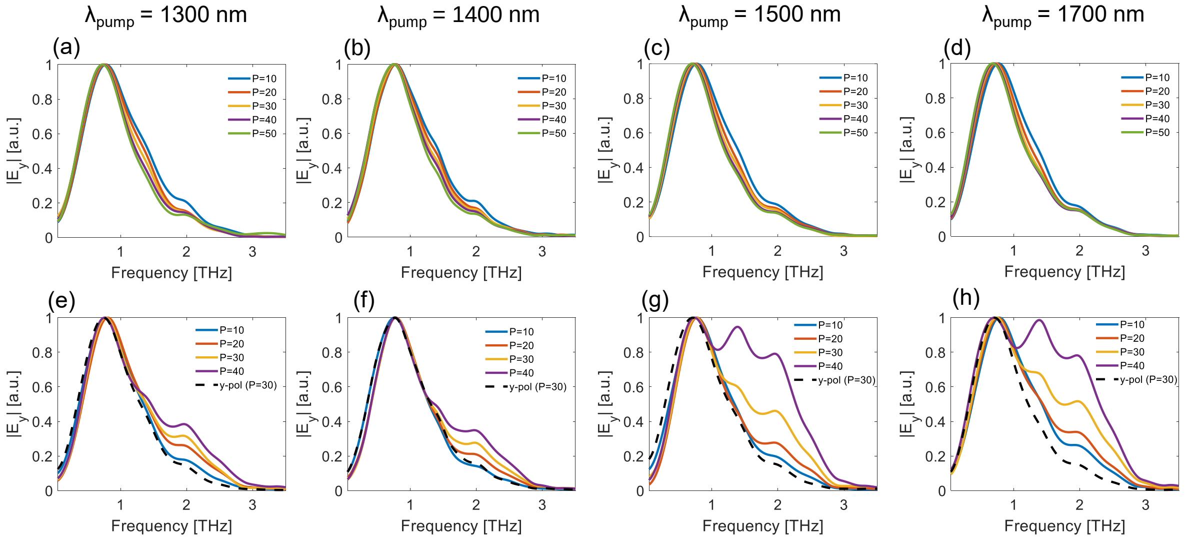

Figure S10: emission spectra at various pumping powers and wavelengths. When pumping the weakly coupled system $E_{in}\hat{y}$ (a-d), and the strongly coupled system - $E_{in}\hat{x}$ (e-h). different colors represent pumping powers of 10mW (blue), 20mW (orange), 30mW (yellow), 40mW (purple), 50mW (green). Static black line in (e-h) shows the static spectra generated when pumping the weakly coupled system ($E_{in}\hat{y}$).

This broadening can be explained by the change in the optical properties of the ITO and gold due to electron and lattice heating. This is shown in Fig. 5 in the main text. Figure S11, shows that pumping the weakly coupled system (y-polarization) does not result in a broadening. The simulations for both x and y polarizations qualitatively agrees with the measurements. The simulated spectrum is calculated as

$$S(\omega) = F(\omega) * T(\omega)$$

where $F(\omega)$ is the spectrum of the generated THz signal calculated from the hydrodynamic model (S4) and $T(\omega)$ is the response function of a 0.5mm thick ZnTe crystal (used as the sampling crystal in our measurements) taken from[3].

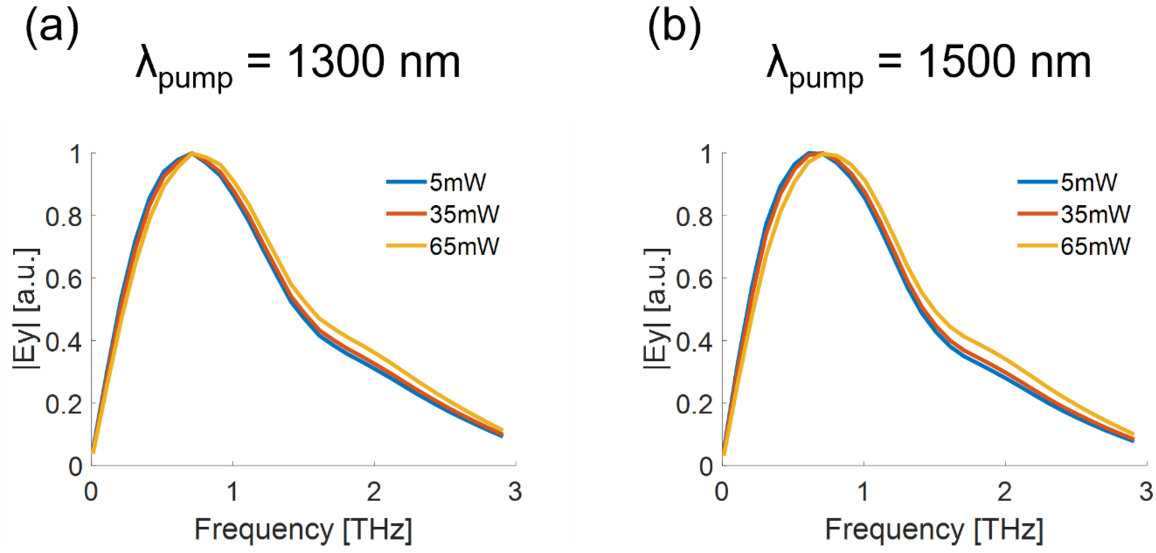

Figure S11: Simulation of generated spectrum at various pumping powers when pumping the weakly coupled system $E_{in}\hat{y}$. a) For pump wavelength of $\lambda_{pump} = 1300nm$ and b) for $\lambda_{pump} = 1500nm$. Colors represent different pumping powers, 5mW (blue), 35mW (orange), 65mW (yellow).

S10) ITO Non-parabolicity parameters

ITO has an isotropic but non-parabolic conduction band, and the dispersion relation can be described by Kane's model of semiconductors as[4]:

$$\frac{\hbar^2 k^2}{2m_0^*} = E + CE^2 \tag{10.1}$$

Where m_0^* is the effective mass at the bottom of the conduction band and C is the non-parabolicity parameter (at C=0 we return to a parabolic band).

Using the electron velocity in the band $v(k) = \frac{1}{\hbar}\frac{\partial E}{\partial k}$, The momentum dependent effective mass $m^*(k)$ is given by [5,6]

$$m^*(k) = \hbar\left[\frac{2}{3}\frac{v(k)}{k} + \frac{1}{3}\frac{dv(k)}{dk}\right]^{-1} \tag{10.2}$$

Which is a weighted average of the more conventional definition of the effective mass $m_c = \hbar\left(\frac{dv}{dk}\right)^{-1}$, and of the alternative definition of the optical effective mass, $m_{opt} = \hbar\left(\frac{v}{k}\right)^{-1}$. In the case of an ideal parabolic band with a linear velocity-momentum relation, all three definitions coincide. However, for a non-parabolic band the definition in (10.2) is crucial to keep the effective mass positive and finite, even when $dv/dk \leq 0$.

Inserting Eq. 10.2 into Eq.10.1 results with an expression for the effective mass:

$$m^* = m_0^* \frac{3(1+2CE)^3}{3+8CE(1+CE)} \tag{10.3}$$

To fit C and m_0^* we follow [7] to find the dependence of the effective mass on the electron concentration and solve for the fermi energy, giving:

$$E_f = \frac{1}{2C}\left(-1 + \sqrt{1 + \frac{2C\hbar^2}{m_0^*}(3\pi^2 N)^{\frac{2}{3}}}\right) \tag{10.4}$$

Substituting Eq.10.4 to Eq.10.3 one can find an expression for the carrier density dependent effective mass:

$$m^* = 3m_0^* \frac{f^{\frac{3}{2}}}{1+2f} \tag{10.5}$$

Where $f = 1 + \frac{2(3\pi^2 N)^{\frac{2}{3}}\hbar^2 C}{m_0^*}$.

Fitting the curve to the measured data in[8] results with the following curve:

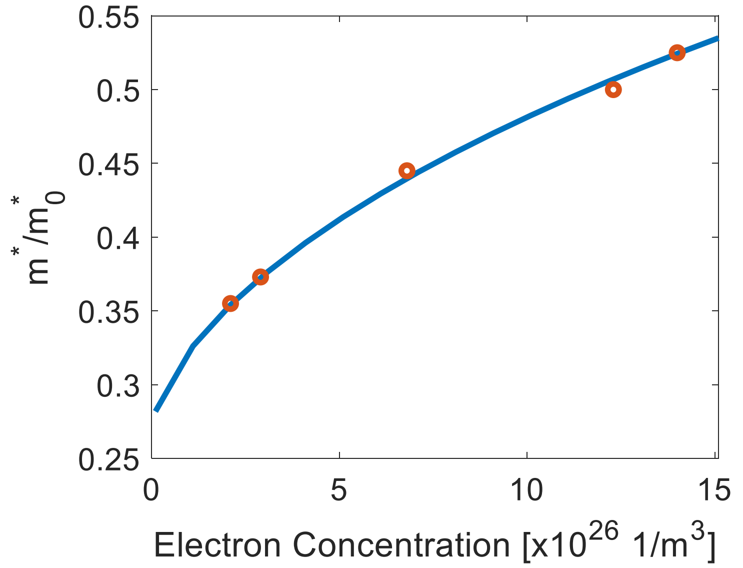

Figure S12: Dependence of the electron effective mass on the carrier density. The blue line is the non-parabolic band fit of Eq. (10.5).

With the fitting parameters:

$$C = 0.212\ eV^{-1}, m_0^* = 0.27 m_e$$

Where m_e is the electron mass.

S11) Temperature dependent effective mass

Electron density (charge) is conserved under intra-band pumping, therefore N is constant and independent on temperature.

$$N = \int_0^\infty \rho(E)\, f_{FD}(E,\mu,T_e)dE \tag{11.1}$$

Where $\rho(E) = \frac{\sqrt{2}}{\pi^2}\left(\frac{m_0^*}{\hbar^2}\right)^{\frac{3}{2}}(E + CE^2)^{1/2}(1 + 2CE)$ is the density of states[9], and $f_{FD} = \left(\mathrm{Exp}\left[\frac{E-\mu}{k_b * T_e} + 1\right]\right)^{-1}$ is the Fermi-Dirac distribution with chemical potential μ, and electron temperature T_e.

The temperature dependent average effective mass is then:

$$\left(m_{avg}^*(T_e)\right)^{-1} = \frac{1}{N}\int_0^\infty \frac{\rho(E) f_{FD}(E,\mu,T_e)}{m^*(E)} dE \tag{11.2}$$

And the temperature dependent plasma frequency then becomes:

$$\omega_p^2 = \frac{Nq^2}{\epsilon_0 m_{avg}^*} \tag{11.3}$$

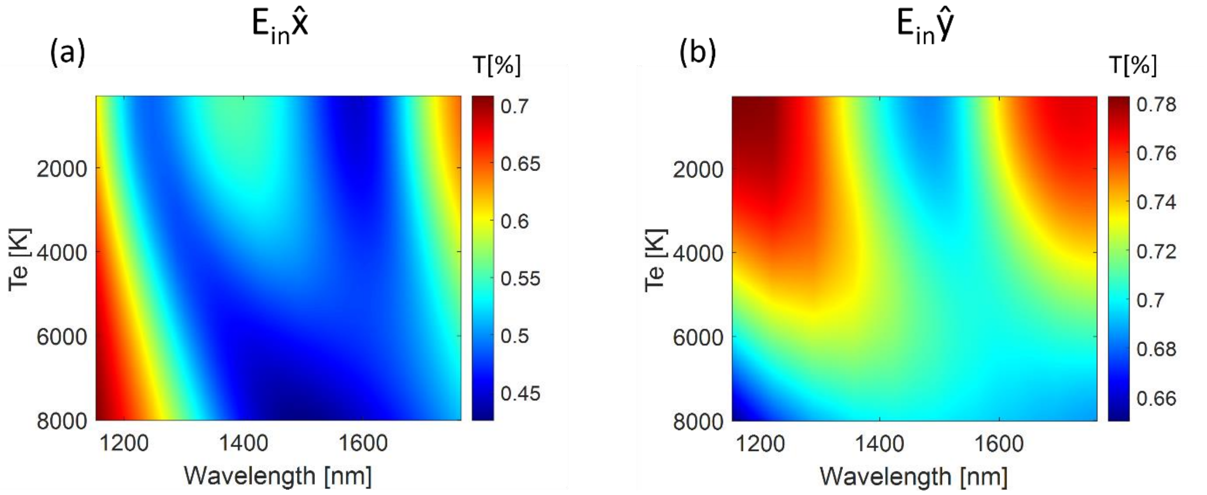

Figure S13: Transmission spectra as a function of electron temperature, for a) the strongly coupled ($E_{in}\hat{x}$) and b) weakly coupled ($E_{in}\hat{y}$) MS, showing strong temperature dependence.

S12) Two temperature model

The ITO film is heavily doped, and therefore the free electron dynamics can be described by the Drude model. The parameters in this model are dependent on the electron and lattice temperatures. To capture these dynamics we use a phenomenological two temperature model [10,11].

According to the delayed two temperature model, the pump light absorbed generates hot electrons with a nonthermal energy distribution which act as a delayed power source. The overall heating dynamics and energy exchange between the electrons and the lattice can be described by a system of coupled differential equations. These equations relate the temperatures of the electrons (T_e) and phonons (T_{lat}) to the time dependent absorbed power density (P_{abs}):

$$\begin{aligned} C_e \frac{\partial T_e}{\partial t} &= \nabla(\lambda \nabla T_e) - G(T_e - T_{lat}) + \Gamma_{ee} U^{NT} \\ C_{lat} \frac{\partial T_{lat}}{\partial t} &= G(T_e - T_{lat}) + \Gamma_{ep} U^{NT} \\ \frac{\partial U^{NT}}{\partial t} &= \frac{1}{C_e} \nabla(\lambda \nabla U^{NT}) - (\Gamma_{ee} + \Gamma_{ep}) U^{NT} + P_{abs} \end{aligned} \tag{12.1}$$

Here, U is the nonthermal energy density, G is the electron-phonon coupling parameter. λ is the thermal conductivity. C_e and C_{lat} are the heat capacities of the electrons and lattice respectively. Γ_{ee} (Γ_{ep}) is the electron-electron (electron-phonon) scattering rate. In these equations we have neglected the phonon diffusion due to its relative small value. Next, we estimate the different parameters that appear in Eq. (11.1) for ITO and for gold.

For ITO, the parameters are calculated as follows:

The temperature dependence of the electron-electron scattering rate was obtained from [12]:

$$\Gamma_{ee} = \frac{\omega^2}{4\pi^2 \omega_p(T_e)} \left[1 + \left(\frac{2\pi k_b T_e}{\hbar \omega} \right)^2 \right] \tag{12.2}$$

The electron-phonon scattering rate which depends on the lattice and Debye temperatures[9]:

$$\Gamma_{ep} = \Gamma_0 \left[\frac{2}{5} + \frac{4 T_{lat}^5}{\Theta_D^5} \int_0^{\Theta_D / T_{lat}} \frac{z^4}{e^z - 1} dz \right] \tag{12.3}$$

Where $\Theta_D = 900K$ is the Debye temperature of the lattice.

The electron heat capacity is given by [13]:

$$C_e(T_e) = \int E \rho(E) \frac{\partial f_{FD}(E, \mu, T_e)}{\partial T_e} dE \tag{12.4}$$

And the lattice heat capacity $C_l = 2.54 * 10^6 \left[\frac{J}{m^3 K}\right]$ is taken to be constant due to relatively small changes in the lattice temperature [9].

The electron phonon coupling coefficient G determines the rate of energy exchange between hot electrons and the lattice and is estimated as [13]:

$$G = \frac{\pi k_b}{\hbar \rho(E_f)} \lambda_{ep} < (\hbar\omega_{ph})^2 > \int \left(-\frac{\partial f_{FD}}{\partial E}\right) \rho(E)^2 dE \tag{12.5}$$

Where λ_{ep} is the electron-phonon mass enhancement parameter, and $< {\omega_{ph}}^2 >$ is an empirical parameter. We use $\lambda_{ep} < (\hbar\omega_{ph})^2 >= 0.0042 eV^2$ [9].

For gold we use the following parameters for the scattering rate[14–16]:

$\Gamma_{ee} = \Delta^{\mathrm{Um}} A \mathrm{T}_{\mathrm{e}}^2$, and $\Gamma_{ep} = BT_l$, where $\Delta^{\mathrm{Um}} = 0.77$ is the Umklapp scattering fraction, with $A = 1.7 * 10^7 \left[\frac{1}{K^2 s}\right]$, and $B = 1.45 * 10^{11} \left[\frac{1}{K^2 s}\right]$.

The phonon heat capacity $C_l = 2.6 * 10^6 \left[\frac{J}{m^3 K}\right]$, was taken as a constant. The electron heat capacity $C_e(T_e)$ and the electron phonon coupling $G(T_e)$ were obtained from [13].

S13) Themo optical response

The permittivity from Eq.1.1 is now also a function of electron and lattice temperatures. For the IR pulse, we can then write the permittivity as:

$$\epsilon(\omega, T_e, T_l) = \epsilon_\infty - \frac{\omega_p^2(T_e)}{\omega^2 + i\omega\Gamma(T_e, T_l)} \quad (13.1)$$

For the ITO film the plasma frequency dependence on temperature is given by Eq.11.3.

For gold, the plasma frequency depends mainly on the lattice temperature due to volume expansion, which alters the density of free electrons in the conduction band [16]:

$\omega_p(T_l) = \sqrt{\frac{q^2}{\epsilon_0 m_{eff}} * \frac{N(T_0)}{1+\beta\Delta T_l}}$, where $\beta = 4.23 * 10^{-5}\left[\frac{1}{K}\right]$, is the thermal expansion coefficient and $m_{eff} \approx m_e$ is the effective electron mass.

The generated THz field is in the semi-static regime, since $\nu_{THz} \sim 10^{12}\left[\frac{1}{s}\right] \ll \Gamma \sim 10^{14}\left[\frac{1}{s}\right]$. Therefore it is convenient to express the permittivity in terms of the conductivity:

$$\epsilon = \epsilon' + i\epsilon'' = \epsilon' + i\sigma(\omega)/\omega \quad (13.2)$$

Where $\sigma(\omega) = \frac{Ne^2}{m_{avg}^* * \Gamma}$. Therefore, the temperature dependent permittivity is given by the following relation:

$$\epsilon_{THz}''(T) = \frac{m_{avg}^*(T_0) * \Gamma(T_0)}{m_{avg}^*(T_e) * \Gamma(T_e, T_l)} \epsilon_{THz}''(T_0) \quad (13.3)$$

References

(1) Ciracì, C.; Poutrina, E.; Scalora, M.; Smith, D. R. Origin of Second-Harmonic Generation Enhancement in Optical Split-Ring Resonators. *Phys. Rev. B* **2012**, *85* (20), 201403. https://doi.org/10.1103/PhysRevB.85.201403.

(2) Sideris, S.; Ellenbogen, T. Terahertz Generation in Parallel Plate Waveguides Activated by Nonlinear Metasurfaces. *Opt. Lett.* **2019**, *44* (14), 3590–3593. https://doi.org/10.1364/OL.44.003590.

(3) Wu, B.; Cao, L.; Zhang, Z.; Fu, Q.; Xiong, Y. Terahertz Electro-Optic Sampling in Thick ZnTe Crystals Below the Reststrahlen Band With a Broadband Femtosecond Laser. *IEEE Trans. Terahertz Sci. Technol.* **2018**, *8* (3), 305–311. https://doi.org/10.1109/TTHZ.2018.2810018.

(4) Kane, E. O. Band Structure of Indium Antimonide. *J. Phys. Chem. Solids* **1957**, *1* (4), 249–261. https://doi.org/https://doi.org/10.1016/0022-3697(57)90013-6.

(5) Secondo, R.; Khurgin, J.; Kinsey, N. Absorptive Loss and Band Non-Parabolicity as a

Physical Origin of Large Nonlinearity in Epsilon-near-Zero Materials. *Opt. Mater. Express* **2020**, *10* (7), 1545–1560. https://doi.org/10.1364/OME.394111.

(6) Khurgin, J. B.; Clerici, M.; Kinsey, N. Fast and Slow Nonlinearities in Epsilon-Near-Zero Materials. *Laser Photon. Rev.* **2021**, *15* (2), 2000291. https://doi.org/https://doi.org/10.1002/lpor.202000291.

(7) Pisarkiewicz, T.; Kolodziej, A. Nonparabolicity of the Conduction Band Structure in Degenerate Tin Dioxide. *Phys. status solidi* **1990**, *158* (1), K5–K8. https://doi.org/https://doi.org/10.1002/pssb.2221580141.

(8) Liu, X.; Park, J.; Kang, J.-H.; Yuan, H.; Cui, Y.; Hwang, H. Y.; Brongersma, M. L. Quantification and Impact of Nonparabolicity of the Conduction Band of Indium Tin Oxide on Its Plasmonic Properties. *Appl. Phys. Lett.* **2014**, *105* (18), 181117. https://doi.org/10.1063/1.4900936.

(9) Alam, M. Z.; Schulz, S. A.; Upham, J.; De Leon, I.; Boyd, R. W. Large Optical Nonlinearity of Nanoantennas Coupled to an Epsilon-near-Zero Material. *Nat. Photonics* **2018**, *12* (2), 79–83. https://doi.org/10.1038/s41566-017-0089-9.

(10) Carpene, E. Ultrafast Laser Irradiation of Metals: Beyond the Two-Temperature Model. *Phys. Rev. B* **2006**, *74* (2), 024301. https://doi.org/10.1103/PhysRevB.74.024301.

(11) Sivan, Y.; Spector, M. Ultrafast Dynamics of Optically Induced Heat Gratings in Metals. *ACS Photonics* **2020**, *7* (5), 1271–1279. https://doi.org/10.1021/acsphotonics.0c00224.

(12) Voisin, C.; Del Fatti, N.; Christofilos, D.; Vallée, F. Ultrafast Electron Dynamics and Optical Nonlinearities in Metal Nanoparticles. *J. Phys. Chem. B* **2001**, *105* (12), 2264–2280. https://doi.org/10.1021/jp0038153.

(13) Lin, Z.; Zhigilei, L. V; Celli, V. Electron-Phonon Coupling and Electron Heat Capacity of Metals under Conditions of Strong Electron-Phonon Nonequilibrium. *Phys. Rev. B* **2008**, *77* (7), 75133. https://doi.org/10.1103/PhysRevB.77.075133.

(14) Parkins, G. R.; Lawrence, W. E.; Christy, R. W. Intraband Optical Conductivity $\ensuremath{\sigma}(\ensuremath{\omega},T)$ of Cu, Ag, and Au: Contribution from Electron-Electron Scattering. *Phys. Rev. B* **1981**, *23* (12), 6408–6416. https://doi.org/10.1103/PhysRevB.23.6408.

(15) Smith, A. N.; Norris, P. M. Influence of Intraband Transitions on the Electron Thermoreflectance Response of Metals. *Appl. Phys. Lett.* **2001**, *78* (9), 1240–1242. https://doi.org/10.1063/1.1351523.

(16) Block, A.; Liebel, M.; Yu, R.; Spector, M.; Sivan, Y.; F, J. G. de A.; N, F. van H. Tracking Ultrafast Hot-Electron Diffusion in Space and Time by Ultrafast Thermomodulation Microscopy. *Sci. Adv.* **2019**, *5* (5), eaav8965. https://doi.org/10.1126/sciadv.aav8965.
